# Colorectal Cancer Screening Participation Among Asian Americans Overall and Subgroups in an Integrated Health Care Setting with Organized Screening

**DOI:** 10.1038/s41424-018-0051-2

**Published:** 2018-09-21

**Authors:** Nirupa R. Ghai, Christopher D. Jensen, Douglas A. Corley, Chyke A. Doubeni, Joanne E. Schottinger, Ann G. Zauber, Alexander T. Lee, Richard Contreras, Theodore R. Levin, Jeffrey K. Lee, Virginia P. Quinn

**Affiliations:** 1 Kaiser Foundation Health Plan, Department of Regional Clinical Effectiveness, 393 East Walnut Street, Pasadena, CA 91188 USA; 2 Kaiser Permanente Northern California, Division of Research, 2000 Broadway, Oakland, CA 94612 USA; 3 Department of Family Medicine and Community Health, The Perelman School of Medicine at the University of Pennsylvania, 51N 39th Street, Andrew Mutch Building, 7th Floor, Philadelphia, PA 19104 USA; 4 Kaiser Permanente Southern California Regional Offices, 393 East Walnut Street, Pasadena, CA 91188 USA; 5 Department of Epidemiology & Biostatistics, Memorial Sloan Kettering Cancer Center, 485 Lexington Avenue, 2063A, New York, NY 10017 USA; 6 Southern California Permanente Medical Group, Kaiser Permanente Woodland Hills, 5601 De Soto Ave, Woodland Hills, CA 91365 USA; 7 Kaiser Permanente Medical Center, 1425 South Main Street, Walnut Creek, CA 94596 USA

## Abstract

**Background:**

Screening reduces colorectal cancer deaths, but <50% of Asian Americans are screening up-to-date according to surveys, with variability across Asian subgroups. We examined colorectal cancer screening participation among Asian Americans overall and Asian subgroups in a large integrated health care system with organized screening.

**Methods:**

Data were electronically accessed to characterize screening in 2016 for Asians overall and subgroups relative to the National Colorectal Cancer Roundtable target of ≥80% screening and compared with non-Hispanic whites. Screening up-to-date was defined as a colonoscopy with 10 years, a sigmoidoscopy within 5 years, or a fecal immunochemical test (FIT) completed in 2016.

**Results:**

Among 436,398 patients, 69,826 (16.0%) were Asian, of whom 79.8% were screening up-to-date vs. 77.6% of non-Hispanic whites (*p* < 0.001). Almost all subgroups met the 80% target: Chinese (83.3%), Vietnamese (82.4%), Korean (82.1%), other Asian (80.3%), Filipino (78.7%), Asian Indian (79.6%), and Japanese (79.0%). Among Asians overall and non-Hispanic whites, 50.6% and 48.4% of members were up-to-date with screening by colonoscopy, and 28.0% and 28.2% were up-to-date by FIT, respectively. Across Asian subgroups, colonoscopy most frequently accounting for being screening up-to-date (range: 47.4–59.7%), followed by FIT (range: 21.6–31.5%).

**Conclusions:**

In an organized screening setting, there were minimal differences in screening participation among Asian subgroups and almost all met the 80% screening target, despite differences in language preference. Screening test type differences across subgroups suggest possible preferences in screening modality, which can inform future research into tailored education or outreach.

## Background

Cancer is the leading cause of death in Asian Americans, with colorectal cancer being the third most common cause of cancer-related deaths^1^. Colorectal cancer deaths can be reduced through screening, and the United States Preventive Services Task Force recommends several screening tests, including high-sensitivity guaiac-based fecal occult blood testing, fecal immunochemical testing (FIT), multi-targeted stool DNA testing, colonoscopy, computed tomography colonography, and flexible sigmoidoscopy with or without FIT^2^.

The National Colorectal Cancer Roundtable set a goal of increasing the colorectal cancer screening rate in the eligible United States population to ≥80% by 2018;^3^ it is estimated that achieving this goal would result in 19% fewer colorectal cancer deaths^4^. However, according to a recent estimate, only 63% of eligible United States residents, and fewer than 50% of some racial/ethnic groups, including Asians, are up-to-date with screening, leading to concern that the 80% target may not be achievable^5^. These findings are consistent with prior population-based surveys which reported low screening rates for Asian Americans and lower screening rates compared with non-Hispanic whites^6–14^. In addition, studies have reported disparities in colorectal cancer screening across Asian American subgroups, but little data exist regarding the influence of organized screening programs on these disparities^10–12^.

Access to health care is strongly associated with participation in colorectal cancer screening^8–15^, and recent studies in medically-insured populations have reported that Asians overall had colorectal cancer screening rates similar to or higher than those of non-Hispanic whites^15,16^. However, in one study, Asian Indians and Filipinos had lower screening rates, while Japanese, Chinese, Korean, and Vietnamese had similar rates to non-Hispanic whites^15^. Low screening participation and disparities across Asian subgroups, if widely observed, may suggest the need for more intensive or tailored approaches to screening.

The aim of the current study was to ascertain colorectal cancer screening participation among Asians overall and Asian subgroups, relative to the 80% screening target and compared to non-Hispanic whites, in a large integrated health care system with organized screening. We hypothesized that with access to organized screening, disparities by race and ethnicity, specifically among Asians and Asian subgroups, would be minimal despite differences in English language proficiency, and that most groups would be at or near the 80% screening target and comparable in their participation to non-Hispanic whites.

## Methods

### Study design, setting, and oversight

This cross-sectional study was conducted among health plan members of Kaiser Permanente Southern California (KPSC), an integrated pre-paid health care system that provides comprehensive medical services to over 4.5 million members. Individuals and their family members enroll in the health plan through their employer, individual plans, or state and federal programs such as Medi-Cal and Medicare. The membership is socioeconomically diverse and broadly representative of the underlying population living in Southern California; in 2010 specifically, Asian/Pacific Islanders comprised 11.8% of the census population in Southern California and 10.1% of KPSC members^17^.

The study was reviewed and approved by the Institutional Review Board at KPSC, which waived the requirement for individual informed consent. The listed authors had sole responsibility for the study design, data collection, decision to submit the manuscript for publication, and drafting of the manuscript. The study was conducted within the National Cancer Institute-funded Population-based Research Optimizing Screening through Personalized Regimens (PROSPR) consortium (U54 CA163262) which conducts multisite, coordinated, transdisciplinary research to evaluate and improve cancer screening processes. Dr. Zauber is supported in part by the Memorial Sloan Kettering Cancer Center Core Grant (P30 CA008748) and Dr. J. Lee is supported by a career development grant from the National Cancer Institute (K07 CA212057).

### Organized colorectal cancer screening program

As previously described^18–20^, each year FIT kits are mailed to eligible health plan members 51–75 years of age who are not screening up-to-date by either a recorded colonoscopy within 10 years or sigmoidoscopy within 5 years. FIT kits are sent annually, regardless of whether prior FIT kits were previously completed, unless a patient tests positive, in which case they are referred for a diagnostic colonoscopy. The goal of the screening program is, primarily through FIT or colonoscopy, to have all eligible members up to date with screening by December 31 of each calendar year, starting the calendar year they turn 51 through 75 years of age, in accordance with the Healthcare Effectiveness Data and Information Set (HEDIS) measurement approach^21,22^. The screening outreach program includes mailed and telephone reminders, and in-reach includes reminders for members attending office or preventive health visits through best practice alerts in the electronic medical record. FIT kits are returned by mail to a regional laboratory and analyzed shortly after the return date. Those with a positive FIT are referred for a follow-up colonoscopy. In lieu of FIT screening, members can request to be screened by colonoscopy.

### Study eligibility criteria

The study population included health plan members 51–74 years of age as of January 1, 2016, enrolled in the health plan for ≥ 1 year prior to study entry (to ascertain screening history), and eligible for colorectal cancer screening (no recorded history of colorectal cancer, colectomy, or inflammatory bowel disease). Asian subgroups were defined using self-identification to the health plan registrars as Filipino, Chinese, Vietnamese, Asian Indian, Japanese, Korean, or other Asian (Lao Loum/Lowland Lao, Lao/Laotian, Malaysian, Nepalese/Nepali, Pakistani, Singaporean/Singapore, Sri Lankan, Taiwanese, Thai). Those self-identifying as non-Hispanic white and who otherwise met the study eligibility criteria served as the comparative group. Race/ethnicity data were available on approximately 95% of active health plan members, and Asian subgroup data were available in approximately 98% of Asians.

### Data sources

Data on patient age, sex, race/ethnicity, membership duration, body mass index (BMI), influenza vaccinations, self-reported language preference, endoscopy procedures (colonoscopy and sigmoidoscopy), and FIT were obtained from electronically-accessible KPSC administrative, clinical, and laboratory databases.

### Colorectal cancer screening outcomes

Colorectal cancer screening status among Asians overall, Asian subgroups, and non-Hispanic whites was determined as of December 31, 2016. Screening up-to-date was defined as having received a colonoscopy or sigmoidoscopy in the prior 10 years (2007–2016) and 5 years (2012–2016), respectively, or completed FIT screening in 2016.

### Statistical analyses

Multivariable log-binomial regression analysis was used to evaluate the association between Asian race/ethnicity (Asians overall and Asian subgroups) and being up-to-date with screening, with non-Hispanic whites serving as the referent group. Prevalence ratios (PR) and 95% confidence intervals (CIs) were adjusted for the following covariates selected a priori: age (continuous); sex; length of health plan membership prior to cohort entry (continuous); BMI (continuous); English language preference (yes/no); and the number of influenza vaccinations in the 3 years before study entry, a surrogate for health care utilization (0, 1, 2, or 3). Tests were two-sided with a significance level of 0.05. BMI and length of membership prior to study entry were the only variables with missing data (7458 and 14,101 patients, respectively); those with missing data were not included in the main adjusted analyses. All analyses were conducted using SAS version 9.4 (SAS Institute Inc., Cary, North Carolina).

## Results

### Characteristics of the population

Among 436,398 individuals in the study population, 16.0% (*n* = 69,826) were Asian, 84.0% (*n* = 366,572) were non-Hispanic white, 44.3% (*n* = 193,299) were 55–64 years of age, and 51.8% (*n* = 226,090) were female (Table 1). Compared with non-Hispanic whites, Asians overall were more likely to be female (*p* < 0.001), have a lower BMI (*p* < 0.001), and have the influenza vaccine annually (*p* < 0.001), they were less likely to prefer English as a language (*p* < 0.001).

**Table 1:** Demographic and clinical characteristics among Asian subgroups, Asians overall, and non-Hispanic whites

	Filipino *n* (%)	Chinese *n* (%)	Vietnamese *n* (%)	Asian Indian *n* (%)	Japanese *n* (%)	Korean *n* (%)	Other Asian *n* (%)	Asians Overall *n* (%)	NH Whites *n* (%)	Total *n* (%)
Total, n (%)	30,201 (43.4)	10,994 (15.8)	7456 (10.7)	6314 (9.1)	4498 (6.5)	4857 (7.0)	5506 (7.9)	69,826 (16.0)	366,572 (84.0)	436,398 (100)
*Age, years*										
50–54	6744 (22.3)	2607 (23.7)	2039 (27.4)	1506 (23.9)	765 (17.0)	947 (19.5)	1099 (20.0)	15,707 (22.5)	75,756 (20.7)	91,463 (21.0)
55–64	13,703 (45.4)	4853 (44.1)	3414 (45.8)	2707 (42.9)	1805 (40.1)	1895 (39.0)	2316 (42.0)	30,693 (44.0)	162,606 (44.3)	193,299 (44.3)
65–74	9754 (32.3)	3534 (32.1)	2003 (26.9)	2101 (33.3)	1928 (42.9)	2015 (41.5)	2091 (38.0)	23,426 (33.5)	128,210 (35.0)	151,636 (34.7)
Female	17,410 (57.7)	6172 (56.1)	3817 (51.2)	3152 (49.9)	2531 (56.3)	2957 (60.9)	3013 (54.4)	39,052 (55.9)	187,039 (51.0)	226,090 (51.8)
Membership, years^a^	13.0 (10.7)	12.9 (11.1)	9.4 (8.7)	11.4 (9.7)	16.5 (13.5)	11.0 (10.2)	11.2 (10.2)	12.4 (10.8)	14.7 (11.5)	14.4 (12.4)
English preferred	27,779 (92.0)	7872 (71.6)	4758 (63.8)	5653 (89.5)	4174 (92.8)	3143 (64.7)	4103 (74.5)	57482 (82.3)	359,875 (98.2)	417,357 (95.6)
*Body mass index, kg/m* ^*2*^										
<18.5	245 (0.8)	364 (3.3)	234 (3.1)	76 (1.2)	139 (3.1)	129 (2.7)	96 (1.7)	1283 (1.8)	3201 (0.9)	4484 (1.0)
18.5–24.9	11,246 (37.2)	6362 (57.9)	4668 (62.6)	2601 (41.2)	1969 (43.8)	2874 (59.2)	2599 (47.2)	32,319 (46.3)	90,671 (24.7)	122,990 (28.2)
25.0–29.9	13,381 (44.3)	3335 (30.3)	2164 (29.0)	2663 (42.2)	1593 (35.4)	1554 (32.0)	1830 (33.2)	26,520 (38.0)	131,772 (35.6)	158,292 (36.3)
≥30.0	5075 (16.8)	770 (7.0)	296 (4.0)	928 (14.7)	726 (16.1)	248 (5.1)	469 (8.5)	8512 (12.2)	134,661 (36.4)	143,173 (32.8)
Missing	254 (0.8)	163 (1.5)	94 (1.3)	46 (0.7)	71 (1.6)	52 (1.1)	512 (9.3)	1192 (1.7)	6267 (1.7)	7459 (1.7)
*Influenza vaccinations* ^b^										
0	5791 (19.2)	2694 (24.5)	1488 (20.0)	1318 (20.9)	985 (21.9)	826 (17.0)	1510 (27.4)	14,612 (20.9)	106,287 (29.0)	120,899 (27.7)
1	4345 (14.4)	1480 (13.5)	920 (12.3)	832 (13.2)	529 (11.8)	659 (13.6)	735 (13.4)	9500 (13.6)	47,812 (13.0)	57,312 (13.1)
2	6499 (21.5)	1958 (17.8)	1421 (19.1)	1272 (20.2)	744 (16.5)	960 (19.8)	1075 (19.5)	13,929 (20.0)	63,206 (17.2)	77,135 (17.2)
3	13,566 (44.9)	4862 (44.2)	3627 (48.7)	2892 (45.8)	2240 (49.8)	2412 (50.0)	2186 (39.7)	31,785 (45.5)	149,267 (40.7)	181,052 (41.5)

^a^Health plan membership duration prior to study entry

^b^Influenza vaccination in the 3 years prior to study entry

The most common Asian subgroups were Filipinos (*n* = 30,201, 43.4%), Chinese (*n* = 10,994, 15.8%) and Vietnamese (*n* = 7456, 10.7%). Asian Indians, Japanese, Koreans, and other Asians (*n* = 4498–6314) comprised 6.5–9.1% of Asians overall (Table 1). English language preference was highest among Japanese (92.8%) and Filipino members (92.0%) and lowest among Vietnamese (63.8%) and Korean members (64.7%).

### Colorectal cancer screening participation

**Fig. 1:**
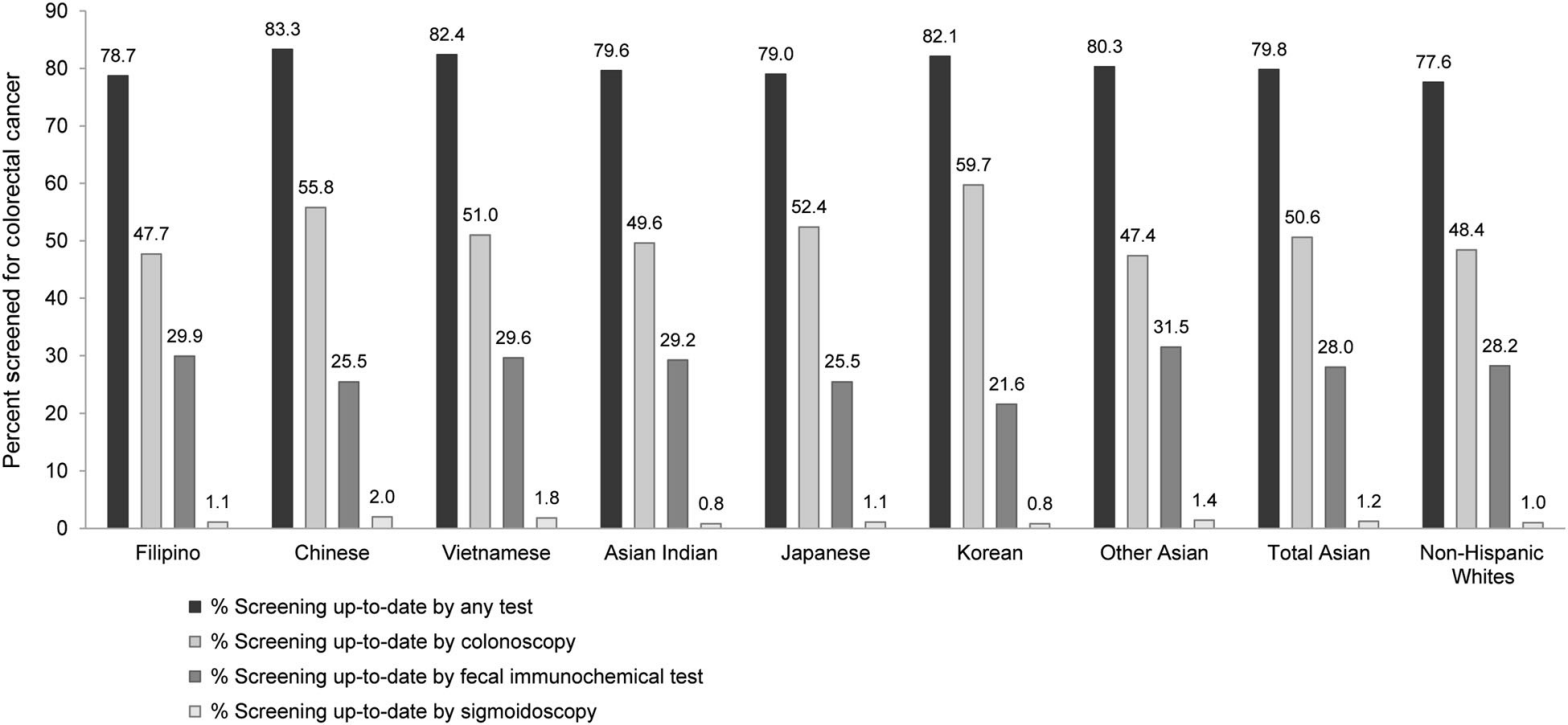
**Colorectal cancer screening participation and test usage among Asian subgroups, Asians overall, and non-Hispanic whites.**

Asians overall were more likely to be up-to-date with colorectal cancer screening than non-Hispanic whites, although the differences were small (79.8% vs. 77.6%, respectively, *p* < 0.001) (Fig. 1). Asian subgroups meeting the ≥ 80% national screening target included Chinese (83.3%), Vietnamese (82.4%), Korean (82.1%), and other Asian members (80.3%), while Filipino (78.7%), Asian Indian (79.6%), and Japanese members (79.0%) fell ≤ 1.3 percentage points short of the target.

**Fig. 2:**
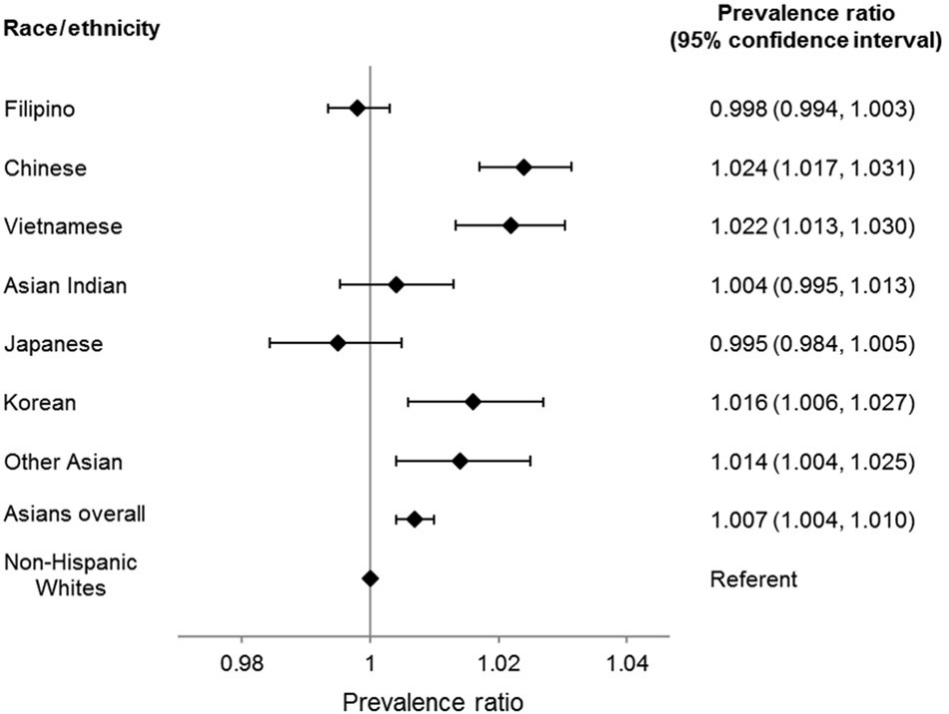
Adjusted prevalence ratios^1,2^ and 95% confidence intervals (CI) among Asian subgroups, Asians overall, and non-Hispanic whites. ^1^Reference group is non-Hispanic whites. ^2^Adjusted for age, sex, length of health plan membership prior to study entry, English language preference, body mass index, and number of influenza vaccines received in the 3 years before study entry

Small differences were also seen in adjusted analyses (Fig. 2). Asians overall (PR 1.007, 95% CI 1.004, 1.010), Chinese (PR 1.024, 95% CI 1.017, 1.031), Vietnamese (PR 1.022, 95% CI 1.013, 1.030), Korean (PR 1.016, 95% CI 1.006, 1.027), and other Asian members (PR 1.014, 95% CI 1.004, 1.025) were slightly more likely than non-Hispanic whites to be up-to-date with screening. The associations between race/ethnicity and screening participation were similar by English vs. non-English language preference for Asians overall and Asian subgroups, and therefore the stratified data are not shown.

### Screening test usage

Among Asians overall and non-Hispanic whites (Fig. 1), 50.6% and 48.4% of members were up-to-date with screening by colonoscopy, respectively, and 28.0% and 28.2% were up-to-date by FIT, respectively. Test choice varied across Asian subgroups, with colonoscopy most frequently accounting for being screening up-to-date (range: 47.4–59.7%), followed by FIT (range: 21.6–31.5%). The largest differences in screening test usage were seen in Korean (59.7% by colonoscopy vs. 21.6% by FIT) and Chinese members (55.8 vs. 25.5%, respectively); the smallest differences were seen in other Asian (47.4% by colonoscopy vs. 31.5% by FIT) and Filipino members (47.7 vs. 29.9%, respectively).

## Discussion

In a large integrated health care setting with organized colorectal cancer screening, primarily FIT outreach and colonoscopy, there were minimal disparities in screening between Asian Americans and non-Hispanic whites, as well as between Asian subgroups, and all groups met (or almost met) the National Colorectal Cancer Roundtable screening target of ≥80%, despite substantial differences between subgroups in English as a preferred language. Colonoscopy was the screening test that most frequently accounted for being up-to-date with screening, followed by FIT, regardless of race, although test choice varied moderately across Asian subgroups.

Our findings contrast with those from prior survey-based studies which reported that Asians and Asian subgroups within California had screening rates well below the 80% screening target, and were less likely to be up-to-date with colorectal cancer screening than non-Hispanic whites^6,9–12,14^, as well as similar reports from national surveys for Asian Americans and Pacific Islanders combined^7,8^. A more recent study examined the colorectal cancer screening status of medically-insured patients in a mixed-payer outpatient health care setting in Northern California between 2012 and 2013, and reported that while all groups were below the 80% screening target, Japanese (68.3% screening up-to-date), Chinese (66.7%), Korean (66.2%), Vietnamese (65.8%), and Filipino patients (59.0%) had higher participation rates than non-Hispanic whites (63.7%), while Asian Indians (45.6%) had lower rates^16^. Our study significantly extends the findings of this latter study, demonstrating that, in a larger integrated health care setting with more recent screening data and a larger population of Asians, an organized screening program was associated with a near absence of disparities in screening across Asian subgroups and achieving screening rates at or near the 80% target is feasible. Our findings are also consistent with a recent report of colorectal cancer screening among 1,746,714 participants across 4 health care systems in the United States, in which Asian Americans and Pacific Islanders combined had higher odds of screening than whites (adjusted odds ratio 1.16, 95% CI 1.15, 1.18)^15^, as well as a study that reported low racial/ethnic disparities in colorectal cancer survival within an integrated health care system in California^23^.

In prior studies, various factors have been linked to low participation in colorectal cancer screening among Asian Americans, including employment status, health insurance access, English language proficiency, health literacy, and length of residency in the United States^24–27^. The high rates of screening and relative lack of disparity in screening participation across Asian subgroups in the current study likely reflect the select nature of the Asian American population studied relative to acculturation factors and barriers to screening. For example, among Asians overall, the average duration of health plan membership was 12.4 years, and most members accessed health insurance through an employer. However, given similar screening rates have not been reported in other settings within California, the high screening participation in the current study may also be related to the integrated and organized nature of cancer screening delivery in this setting, including outreach and in-reach initiatives aimed at reducing barriers to screening, particularly annual FIT outreach. Within Kaiser Permanente Northern California (KPNC), where this has been looked at extensively, screening rates rose from approximately 39% in 2000-2005, to 66% in 2008 (when the implementation of programmatic annual FIT screening was completed), to > 80% starting in 2011; the sharp increase in screening paralleled an increase in the uptake of FIT^28^. These findings are similar to what was reported among racial/ethnic groups within KPNC, including Asians/Pacific Islanders in aggregate^20^. Screening rates within KPSC mirror those of KPNC, although they are unpublished. Beyond the annual mailing of FIT kits, patients could opt instead for endoscopy screening (primarily colonoscopy); offering multiple screening options may increase screening uptake^29,30^. Through electronic medical record prompts, all physicians, not just primary care providers, were informed of a patient’s screening status at every health care visit, and FIT kits were made available to patients not-up-to-date with screening despite mailed outreach. Also, tailored outreach materials (e.g., in different languages) and interpreters were utilized to explain colorectal cancer screening to patients, and patient language preference was matched to the language spoken by the physician when possible; these efforts at tailoring outreach and in-reach may help explain why English language preference did not modify the association between race/ethnicity and the likelihood of being screening up-to-date as has been reported in other studies^14,16,24–27^. Finally, screening rates across medical facilities were tracked and reported monthly as performance improvement measures. Attention to these system-, provider-, and patient-level factors may enhance more uniform access to screening for patients, despite differences in patient demographics.

The finding of Asian subgroup variation in screening test usage, despite standardized outreach procedures, suggests there may be cultural differences in the acceptance of different screening modalities. Other studies have reported racial/ethnic disparities in screening test usage. In a cluster randomized trial of different colorectal cancer screening strategies, nonwhites were more likely to complete fecal occult blood testing than whites, whereas whites were more likely to complete colonoscopy than nonwhites^30^. Similar findings were reported for Asian/Pacific Islanders compared to non-Hispanic whites within Kaiser Permanente Northern California health plan members^20^. Differences in screening test usage between Asian American subgroups has not been extensively explored, but may reflect differences in barriers related to discussing health concerns (Wong et al. 2005) and attitudes about prioritizing preventive health care (Jung et al. 2017)^10,31^. Further research to identify the factors driving these differences in screening modality usage may allow more tailored education or outreach.

Strengths of the present investigation include the large sample of Asians overall and Asian subgroups, and the comprehensive capture of colorectal cancer screening tests, including FIT from the laboratory database, endoscopy procedures from procedure codes, and claims data for procedures performed outside the health system; in contrast, survey-based studies relying on self-reports of screening are subject to recall and social desirability biases, low response rates, and overestimation of actual screening completion. A limitation of the study is that results may not be generalizable to health care settings with limited ability to deliver colorectal cancer screening or to individuals with limited access to health care. In addition, we did not have access to some known predictors of screening, such as employment status, English language proficiency, health literacy, and length of residency in the United States, which may contribute to omitted variable bias and error in the regression estimates.

In conclusion, in contrast to survey data suggesting that <50% of Asian Americans are up-to-date with colorectal cancer screening, with variation across Asian subgroups, in a setting with organized colorectal cancer screening, there was minimal variation in screening participation and all subgroups we studied were close to or exceeded the 80% screening target set by the National Colorectal Cancer Roundtable. While this study did not test an intervention, the findings suggest the potential for organized screening programs to achieve high screening rates. Further research into the variation in screening test usage across Asian subgroups may allow more tailored education or outreach approaches.

## Study Highlights

### What is current knowledge

- National surveys report that < 50% of Asian Americans are up-to-date with colorectal cancer screening, with variability across Asian subgroups.
- The National Colorectal Cancer Roundtable screening target for the United States is ≥ 80% by 2018.

### What is new here

- In an organized colorectal cancer screening program, approximately 80% of Asians overall were screening up-to-date in 2016, with minimal variability across subgroups.
- Test use (colonoscopy vs. fecal immunochemical testing) varied across Asian subgroups.
- Differences in screening test usage across Asian subgroups may require tailored education or outreach.
